# 708. Evaluation of Fungal Culture versus Bacterial Culture for the Identification of Various Mold Species

**DOI:** 10.1093/ofid/ofab466.905

**Published:** 2021-12-04

**Authors:** Erin Su, Rosemary She

**Affiliations:** 1 Keck School of Medicine, chino hills, California; 2 University of Southern California, Los Angeles, CA

## Abstract

**Background:**

Invasive mold infections are challenging to diagnose and in part relies on fungal cultures. A large proportion of mold isolates are recovered on routine bacterial cultures in our medical center, thus we sought to define the utility of bacterial versus fungal cultures for isolation of mold from clinical specimens.

**Methods:**

Routine bacterial and fungal culture results from wound, tissue, body fluid, and respiratory specimens from Jan 2019-Dec 2020 from Keck Medical Center of USC (Los Angeles, CA) were retrospectively reviewed. Cases were excluded if specimens were collected specifically for dermatophyte recovery or for blood culture. Cultures in which mold, including dimorphic fungi, were isolated were included in the evaluation.

**Results:**

Mold was isolated from 612 specimens from 408 patients, with recovery from 329 bacterial and 450 fungal cultures. Among the 329 bacterial cultures, fungal cultures were not requested in 119 (36.2%) while the remaining 210 had concurrent fungal cultures which recovered mold in 167 cases (79.5%). Of 450 fungal cultures recovering mold, a corresponding bacterial culture was performed in 445, isolating mold in 181 (38.8%) of these cases. Two or more molds were found in 28 fungal cultures and in 5 bacterial cultures. Of positive specimens with both fungal and bacterial cultures performed (n=488), mold was isolated in fungal cultures in 446 (91.4%) and in bacterial cultures in 209 (42.9%) (Table).

Yield of molds in 488 specimens with concomitant bacterial and fungal cultures

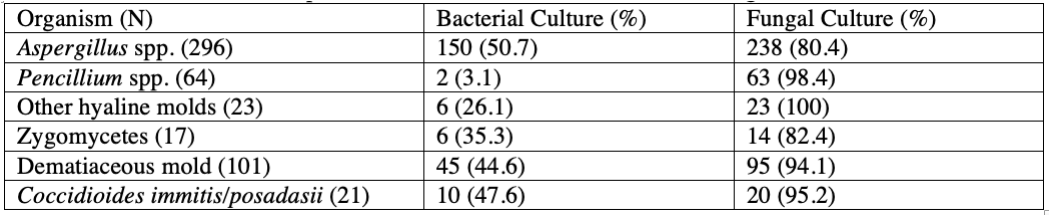

**Conclusion:**

Although a significant number of molds are recovered in routine bacterial cultures, over half would be missed without concomitant fungal cultures. Conversely, recovery of clinically relevant mold species was optimal when both bacterial and fungal cultures were requested on a specimen. This may be related to increased specimen sampling and incubation conditions allowing for broader organism recovery.

**Disclosures:**

**All Authors**: No reported disclosures

